# Predicting Response of Severe Aplastic Anemia to Rabbit-Antithymocyte Immunoglobulin Based Immunosuppressive Therapy Combined With Eltrombopag

**DOI:** 10.3389/fimmu.2022.884312

**Published:** 2022-05-26

**Authors:** Ruixin Li, Jiongtao Zhou, Zhengyuan Liu, Xi Chen, Qiqiang Long, Yan Yang, Shengyun Lin, Jinsong Jia, Guangsheng He, JianYong Li

**Affiliations:** ^1^Department of Hematology, The First Affiliated Hospital of Nanjing Medical University, Jiangsu Province Hospital, Collaborative Innovation Center for Cancer Personalized Medicine, Nanjing, China; ^2^Department of Hematology, The Second Hospital of Nanjing, Nanjing, China; ^3^Department of Hematology, The First Bethune Hospital of Jilin University, Changchun, China; ^4^Department of Hematology, Zhejiang Province Hospital of Traditional Chinese Medicine, The First Affiliated Hospital of Zhejiang Chinese Medical University, Hangzhou, China; ^5^Department of Hematology, Peking University People’s Hospital, Peking University Institute of Hematology, Beijing, China

**Keywords:** eltrombopag, intensive immunosuppressive therapy, rabbit antithymocyte immunoglobulin, efficacy, severe aplastic anemia

## Abstract

Addition of eltrombopag (E-PAG) to intensive immunosuppressive therapy (IST) contributes to restoring hematopoiesis in patients with severe aplastic anemia (SAA). Used at relatively low doses in the East Asian population, the efficacies of E-PAG and the predictors for efficacy are not clear. We conducted a retrospective, multicenter study to analyze the efficacy and the possible predicting factors at 6 months in 58 adult SAA patients with rabbit ATG-based IST and E-PAG. The response rate and complete response rate at 6 months were 76% and 21%, respectively. The baseline reticulocyte percentage [area under a curve (AUC)=0.798, 95% confidence interval (CI) 0.640-0.956, *P*=0.006], absolute reticulocyte count (ARC) (AUC =0.808, 95%CI 0.647-0.970, *P*=0.004), red cell distribution width – coefficient of variation (RDW-CV) (AUC=0.722, 95%CI 0.494-0.950, *P*=0.040), and absolute lymphocyte count (ALC) (AUC=0.706, 95%CI 0.522-0.890, *P*=0.057) were highly predictive of response at 6 months. The tipping values of reticulocyte percentage, ARC, RDW-CV, and ALC were 0.45%, 7.36×10^9^/L, 11.75%, and 1.06×10^9^/L, respectively. The sensitivity and specificity of reticulocyte percentages were 81.6% and 66.7%; ARC were 86.8% and 66.7%, RDW-CV were 94.7% and 55.6%; ALC were 55.3% and 88.9%. At a median follow-up of 15.5 months, the 2-year cumulative overall survival was 92%. The baseline reticulocyte percentage, ARC, RDW-CV, and ALC were potential factors in predicting a favorable effect of rabbit-ATG based IST plus E-PAG in SAA patients of East Asia (ChiCTR2100045895).

**Clinical Trial Registration:**

http://www.chictr.org.cn/edit.aspx?pid=125480&htm=4, identifier ChiCTR2100045895.

## Introduction

Severe aplastic anemia (SAA) is an immune bone marrow failure (BMF) syndrome mainly mediated by autoreactive T lymphocytes (1, 2). Intensive immunosuppressive therapy (IST) is recommended for patients who are not suitable for hematopoietic stem cell transplantation (HSCT). About two-thirds of patients have a response to IST compounded by antithymocyte immunoglobulin (ATG) and cyclosporin A (CsA) (3, 4).

By binding to the transmembrane domain of the thrombopoietin receptor, eltrombopag (E-PAG) blocks the inhibitory effect of interferon-γ (IFN-γ), stimulating the hematopoiesis recovery (5). E-PAG could restore trilineage hematopoiesis in refractory/relapse SAA (6–8). When added to standard horse ATG (h-ATG) plus cyclosporine, E-PAG resulted in better efficacy in untreated patients with SAA (9).

With different pharmacokinetics in different populations, the recommended dosage of E-PAG is 75mg/d in the East Asian population (9). It is also reported that h-ATG has better efficacy than rabbit ATG (r-ATG) (10). However, there is no h-ATG in the mainland of China. Therefore, it is necessary to investigate the efficacy of E-PAG at a dose of 75 mg/d combined with r-ATG based IST in East Asian population. It is also important to identify patients with a high probability of response, since E-PAG is expensive.

Hence, we retrospectively analyzed the efficacy and the possible predicting factors in 58 adult patients with SAA who received r-ATG-based IST combined with E-PAG in the China Eastern Cooperation Group for Anemia (CECGA).

## Materials and Methods

### Patients

From February 2018 to December 2020, patients 18 years of age or older who had previously untreated SAA were eligible for CECGA which has included the First Affiliated Hospital of Nanjing Medical University, the First Bethune Hospital of Jilin University, Peking University People’s Hospital, Zhejiang Provincial Hospital of Chinese Medicine, and Tongji Hospital of Tongji Medical College of Huazhong University of Science and Technology (ChiCTR2100045895). The diagnosis referred to the modified Camitta criteria (3). The exclusion criteria included congenital hematopoietic failure, clinically classic paroxysmal nocturnal hemoglobinuria (PNH), and myelodysplastic syndrome (MDS). Next generation sequencing (NGS) for targeting myeloid malignancy gene mutations, including in *DNMT3A, BCOR, ASXL1, TET2, RUNX1, TP53, U2AF1, SRSF2, IDH1, IDH2, JAK2, KRAS, MPL, NRAS, PIGA, SETBP1, SF3B1, SH2B3, ZRSR2, CEBPA, FLT3, KIT, NPM1, GATA2, MLL, PDGFRA, PHF6, WT1, EZH2, ETV6, CSF3R, CBL, CALR*, and *BCORL1*, was conducted on the Illumina (Solexa) second-generation sequencing technology platform. The study protocol was approved by the ethics committee of each participating hospital and conformed to the recently revised Declaration of Helsinki.

### Therapeutic Regimen

The r-ATG was administered intravenously at a dose of 3.5mg per kilogram of body weight per day for 5 consecutive days. Oral CsA was administered at a dose of 3-5mg per kilogram of body weight per day with a minimum concentration of 150-200ng/ml, where the dosage could be modulated on the basis of drug concentration and unwanted side effects. E-PAG was administered orally at a dose of 75mg per day for at least 6 months. If the count of platelet (PLT) was higher than 200×10^9^/L or severe adverse events (AEs) presented, E-PAG would be reduced or discontinued (11). The dosage would be maintained at 75mg/d in patients holding the potential to respond completely when fluctuating level of PLT count was between 100×10^9^/L and 200×10^9^/L.

### Response Criteria

Complete remission (CR) is defined as the absolute count of neutrophil (ANC) >1.0×10^9^/L, hemoglobin (Hb) >100g/L, the count of PLT >100×10^9^/L, and not requiring transfusion. The criteria for the partial remission (PR) are transfusion independence, with ANC >0.5×10^9^/L, Hb >80g/L, and PLT count >20 × 10^9^/L, but is insufficient for CR. Non-remission (NR) is defined as failure to meet any of the above response criteria. Patients who did not complete 6 months of initial IST due to death were counted as non-responders. Relapse is regarded as a decrease in peripheral blood counts to values either requiring transfusions or needing a second course of IST or undergoing HSCT (9). It is inevitable that the blood cell counts of many patients are diminished slightly following CsA dosage diminution, but blood counts rise once again while the dosage of CsA is increased to the previous treatment dose. The aforementioned condition is not perceived as relapse.

The blood count, hepatic, and renal function are examined at least every 2 weeks after treatment initiation, while efficacies are evaluated at 3, 6, and 12 months after the start of treatment, and then the patients are followed up at least every 3 months.

### Statistical Analysis

Statistical analyses were performed using an SPSS 25.0 software package. Independent-samples t-test and Mann-Whitney U test were used to compare numerical variables. The chi-square test was used to compare categorical variables. The binary logistic regression model was used to assess independent predictors of responses, while the receiver operating characteristic (ROC) curve was used to evaluate the efficacy predictors of E-PAG. Variables with *P*<0.1 in the univariate analysis were included in the multivariate analysis. The prediction bounds of each index were taken at the maximum of Youden index. The sensitivity and specificity, as well as area under the curve (AUC), were calculated. P<0.05 is defined as a statistically significant difference.

## Results

### Patient Characteristics

Fifty-eight patients aged 18-74 years (median, 42.5 years) were treated with rabbit ATG-based IST combined with E-PAG, in which 44 patients responded and 14 patients did not respond at 6 months after treatment. The clinical characteristics of the patients are summarized in **Table 1**. No significant inter-group differences were noted in gender, age, the time between diagnosis and treatment, and some baseline laboratory characteristics, such as counts of red blood cells, Hb, PLT, RDW-CV, ferritin, T cell subsets (including CD3, CD4, CD8, and regulatory T cell), and the prevalence of PNH clone. The responders showed significantly higher ANC (*P*=0.05), ALC (*P*=0.043), reticulocyte percentage (*P*=0.002), absolute reticulocyte count (*P*=0.001), ratio of SAA (*P*=0.036), and lower rate of infection before treatment (*P*=0.031) compared to non-responders (**Supplementary Table S1**).

**Table 1:** Baseline clinical and laboratory characteristics^*^.

Characteristics	Responders (n=44)	Non-responders (n=14)	Total (n=58)
Age, years	38 (18-71)	50.5 (24-74)	42.5 (18-74)
Male sex	24 (55%)	4 (29%)	28 (48%)
Severity: SAA	34 (77%)	6 (43%)	40 (69%)
Time from diagnosis to treatment, d	16 (1-741)	18 (3-1161)	16 (1-1161)
RBC, ×10^12^/L	1.9 (1.2-4.1)	2.0 (1.0-2.5)	1.9 (1.0-4.1)
Hb, g/L	63 (44-128)	60 (37-85)	62 (37-128)
PLT, ×10^9^/L	9 (2-40)	6 (4-19)	8 (2-40)
ANC, ×10^9^/L	0.4 (0-4.0)	0.1 (0-0.7)	0.4 (0-4.0)
ALC, ×10^9^/L	1.1 (0.1-2.8)	0.8 (0.1-2.0)	1.1 (0.1-2.8)
ARC, ×10^9^/L	17.74 (0.92-97.92)	5.80 (0.45-30.35)	14.53 (0.45-97.92)
Reticulocyte, %	0.95 (0.06-5.73)	0.39 (0.02-1.51)	0.76 (0.02-5.73)
RDW-CV, %	14.8 (11.2-23.4)	12.6 (10.8-19.9)	14.8 (10.8-23.4)
CD3, %	81.0 (27.6-93.3)	84.2 (67.0-89.9)	82.2 (27.6-93.3)
CD4, %	43.0 (11.4-74.9)	49.5 (34.8-67.5)	44.8 (11.4-74.9)
CD8, %	27.3 (8.9-65.6)	21.7 (14.8-41.0)	26.8 (8.9-65.6)
Treg, %	1.9 (0-11.7)	1.0 (0-5.6)	1.8 (0-11.7)
Ferr, ng/ml	579.2 (47.0-2377.0)	1015.0 (156.8-5597.5)	605.4 (47.0-5597.5)
PNH clone	2 (5%)	2 (14%)	4 (7%)
Infection before IST	14 (32%)	9 (64%)	23 (40%)

^*^Data represented the number (%) or median (range). SAA, severe aplastic anemia; RBC, red blood cell; Hb, hemoglobin; PLT, platelet; ANC, absolute neutrophil count; ALC, absolute lymphocyte count; ARC, absolute reticulocyte count; RDW-CV, red cell distribution width - coefficient of variation; Treg, regulatory T cell; Ferr, ferritin; PNH, paroxysmal nocturnal hemoglobinuria; IST, immunosuppressive therapy.

### Response

The overall response rates (ORR) were 64%, 76%, and 85% (44 of 52) at 3, 6, and 12 months, respectively, with the CR rates 19%, 21%, and 29% (15 of 52). The median time to the first response was 2 months (0.5 month to 12 months) and the median time to CR was 6 months (2 months to 23 months).

In univariate analysis, patients with vSAA (*P*=0.020), lower reticulocyte percentage (*P*=0.021), and infection before treatment (*P*=0.036) had a lower probability of response at 6 months (**Table 2**). However, none of these factors or others (including age, sex, time between diagnosis and treatment, ALC, and ANC) was found associated with the efficacy at 6 months on multivariate analysis (**Table 2**).

**Table 2:** Factors related to the efficacy at 6 months after IST with univariate and multivariate analysis.

Category and variable	*Value P*	
	Univariate	Multivariate
Age (years)	0.090	0.26
Gender Male vs. Female	0.098	0.148
Severity SAA vs. vSAA	0.020	0.70
Time from diagnosis to treatment	0.35	0.22
Infection before IST	0.036	0.51
ANC before IST	0.076	0.82
ALC before IST	0.060	0.37
Reticulocyte (%)	0.021	0.131

SAA, severe aplastic anemia; vSAA, very severe aplastic anemia; IST, immunosuppressive therapy; ANC, absolute neutrophil count; ALC, absolute lymphocyte count; IST, immunosuppressive therapy.

### Predictors of the Effect

ROC curve was used to evaluate the factors predicting the efficacy of E-PAG if the indexes had *P-*value lower than 0.1 in univariate studies or had a possible impact on the efficacy.

**Figure 1:**
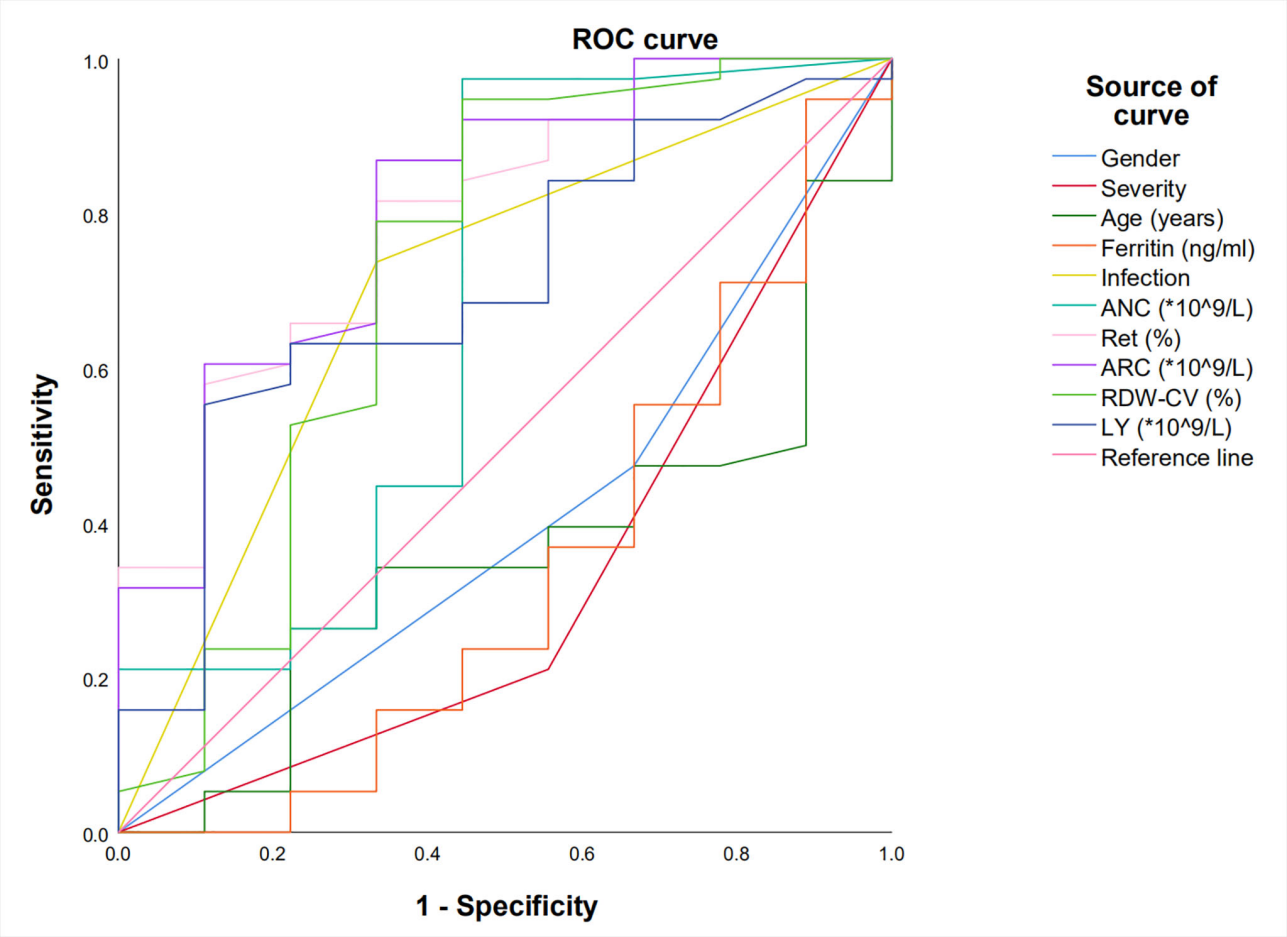
Receiver operating characteristic (ROC) curve was used to predict factors influencing the effect of E-PAG at 6 months. Reticulocyte percentage, ARC, RDW-CV and ALC with AUC more than 0.7.

The reticulocyte percentage, ARC, RDW-CV, and ALC were with an AUC of 0.789 [95% confidence interval (CI) 0.640-0.956, P=0.006], 0.808 (95%CI 0.647-0.970, P=0.004), 0.722 (95% CI 0.494-0.950, P=0.040), and 0.706 (95% CI 0.522-0.890, P=0.057), respectively. The tipping values of reticulocyte percentage, ARC, ALC, and RDW-CV were 0.45%, 7.36×10^9^/L, 1.06×10^9^/L, and 11.75%, respectively, at the maximum of the Youden index. The sensitivity and specificity of reticulocyte percentages were 81.6% and 66.7%; ARC were 86.8% and 66.7%, RDW-CV were 94.7% and 55.6%; ALC were 55.3% and 88.9% (**Figure 1**, **Table 3**). The pretreatment factors including age, gender, severity, ferritin, infection, and ANC were not found to be predictive of the efficacy of E-PAG.

**Table 3:** Prediction performance of the ROC analysis model.

Index	AUC	Sensitivity(%)	Specificity(%)	The optimum critical value	Youden index
Reticulocyte percentage	0.798	81.6	66.7	0.45%	0.483
ARC	0.808	86.8	66.7	7.36×10^9^/L	0.535
RDW-CV	0.722	94.7	55.6	11.75%	0.503
ALC	0.706	55.3	88.9	1.06×10^9^/L	0.442

ROC, receiver operating characteristic; AUC, area under a curve; ARC, absolute reticulocyte count; RDW-CV, red cell distribution width - coefficient of variation; ALC, absolute lymphocyte count.

### Clone Evolution and Survival

The median follow-up time was 15.5 months (1 month to 35 months), one patient relapsed at 13 months after treatment, and still required transfusions at 33 months after IST. One patient achieving PR at 3 months turned into PNH at 12 months.

All patients underwent routine chromosome examinations prior to therapy. Cytogenetic karyotype data of 40 patients were available and the results were normal.

Forty-eight patients whose samples were evaluated for gene mutations by NGS at baseline, in which four patients had detectable somatic mutations (one mutation of each), including *DNMT3A* (three patients) and *MPL* (one patient). The variant allele frequency (VAF) was 5.1%, 4.9%, 2.9%, and 5.9%, separately. Three patients achieved CR, and one patient achieved PR. One patient with *DNMT3A* relapsed at 13 months after PR as mentioned above (**Supplementary Table S2**). When this patient relapsed, the *DNMT3A* was present with VAF of 11.8% and the chromosome was normal. The other three patients remained in continuous remission with no signs of transformation to MDS and acute myelogenous leukemia, and thus the examinations of cytogenetic karyotype and NGS were not performed. Data of NGS were available for 20 patients during follow-up, new additional mutations were acquired in four patients including *DNMT3A* (three patients) and *BCOR* (one patient) with VAF of 3.6%, 4.3%, 8.0%, and 16.5% respectively. All four patients were responders.

The cumulative overall survival (OS) was 92%. Four patients died within 2 years, in which two patients died early due to pulmonary infection and cerebral hemorrhage, respectively. One patient died due to cerebral hemorrhage at 9 months and one patient died at 13 months. In addition, these patients had lower reticulocyte percentage, ARC, RDW-CV, and ALC than the optimum critical values, aside from one patient with RDW-CV of 12.8% and one with RDW-CV of 19.5%.

Because of the small size of the study cohort and dead patients, the correlation of reticulocyte percentage, ARC, RDW-CV, as well as ALC with OS, was not evaluated.

## Discussion

In a cohort study at the National Institutes of Health (NIH), patients with newly diagnosed SAA received h-ATG-based IST and E-PAG. It was reported that the ORR was 94% and the CR rate was 58% at 6 months when patients were treated with E-PAG from day 1 to 6 months after IST, which was higher than in previous studies (9, 10, 12). In a multicenter prospective randomized controlled study in Europe (RACE), previously untreated SAA patients were administered with h-ATG-based IST plus E-PAG; the CR rate and ORR at 6 months were 32% and 68%, respectively (13).

Nonetheless, Assi, et al. reported that IST combined with E-PAG did not improve outcomes in a prospective randomized controlled study. However, in the study of Assi et al., IST was not used simultaneously with E-PAG, and the median age of patients was also older, reaching 60 years old. Additionally, the ANC and reticulocyte percentages were lower in the IST and E-PAG cohorts than in the IST alone cohort (14).

The efficacy of IST was correlated with age. In a large retrospective study, r-ATG-based IST was used to treat 955 AA patients (15). Through multivariate analysis, age was associated with efficacy and survival (15). In different age brackets (0–20, 21–40, 41–60, >60 years), the response rates were 55%, 52%, 47%, and 38% at 6 months (15). Relatively, the median age was 32 years old and 55 years old in studies of NIH and RACE (9, 13). The median age of the patients in our study was 42.5 years, which also appeared to be older than the patients in the NIH study. The study had shown that h-ATG was better than r-ATG in the treatment of SAA (10). Since h-ATG is not yet available in China, we used r-ATG-based IST in this study. In our study, the ORR and CR rates at 6 months were 76% and 21%, respectively, which is lower than those in the NIH study, probably due to differences in age and ATG. The recommended dosage of E-PAG in the East Asian population is 75mg/d, which is half of that for the non-Asian population. The dose of E-PAG was 75mg/d in our study, so whether this is one of the reasons for the difference in efficacy remains to be determined.

As a new therapeutic strategy, the influencing factors and predictors of efficacy of E-PAG combined with IST are still not clear. Fattizzo et al. found in the study of 49 AA patients that nonsevere aplastic anemia (NSAA) (P < 0.005), lower percentage of bone marrow lymphocytes (P < 0.05), and PNH clone (P < 0.05) were related to the overall efficacy, but multivariate analysis was not used to predict the efficacy (16). Zaimoku et al. retrospectively reported that patients with SAA treated with IST plus E-PAG had better efficacy at 6 months when they had higher ANC (*P*=0.00027) and higher ARC (*P*=0.0009), and a lower TPO level (*P*=0.0037). In addition, in patients over 10 years old, patients with higher ALC had better efficacy (*P*<0.05). In the multivariate logistic regression analysis, the independent predictive factors of the ORR in IST combined with the E-PAG group were ARC (*P*=0.018) and TPO (*P*=0.039) (17).

In our study, the Mann-Whitney U test and the chi-square test were used to analyze baseline characteristics. The baseline severity, infection, ALC, ARC, and reticulocyte percentage were significantly different between responders and non-responders. Univariate logistic regression analysis showed that severity, reticulocyte percentage, and infection before treatment were related to efficacy at 6 months. ROC curve showed that reticulocyte percentage, ARC, RDW-CV, and ALC could predict responses at 6 months.

In the ATG-based era, Scheinberg et al. indicated that ALC and ARC could predict the effect on adult SAA patients (18). The recovery in patients with SAA after IST is due to the elimination of autoreactive T cell targeting hematopoietic stem cells and progenitor cells in the bone marrow, and then hematopoiesis is improved. A higher baseline ALC might indicate the presence of numerous autoreactive T cells that need to be eliminated, and thus IST could achieve better results. ARC could help clinically assess bone marrow function since the robust recovery of ARC following IST could predict long-term survival (19). RDW was used to evaluate the scope of RBC volume. The width of RDW resulted from immature RBC released into the peripheral blood circulation (20). Reticulocyte percentage, ARC, and RDW-CV all reflect the residual hematopoietic status of bone marrow. E-PAG has been used to improve hematopoietic repopulation, while the mechanism is still ambiguous. Small molecule compound E-PAG, exempt from the interference of various inflammatory factors in the blood, promoted the proliferation of hematopoietic precursor cells by entering the “niche” of hematopoietic stem/progenitor cells directly (5, 21). It has been reported that E-PAG expanded the size of CD34+ cells and hematopoietic multipotent progenitor cells (22). The percentage of reticulocyte, ARC, and RDW-CV predicting the efficacy also suggested that E-PAG could improve the bone marrow residual hematopoietic function.

The presence of PNH clones at baseline was considered to be a marker of favorable response to IST in adults (23). However, the correlation has not been confirmed in NIH and Japanese cohorts of children (18, 24), nor in our study.

In our study, CD3^+^, CD8^+^ cells, and Treg failed to predict the hematological response of IST combined with E-PAG, suggesting that the bone marrow targets of immune attack in SAA remained elusive. Some laboratory findings reflect the pathophysiology of SAA, such as the amounts of activated T-cell producing IFN-γ, telomere length, and telomerase genes mutations, and the presence of a small number of aneuploid myeloid cells may be prognostic factors (25–27).

As E-PAG improved the ORR and CR rate of IST in SAA, it broadened the risk-benefit analysis of HSCT and ATG. The modality of therapies could be better assessed when the response to IST could be predicted in SAA. Salvage therapies might be designed early post-IST. For patients with a low probability of response who do not achieve a hematologic response within 3 months, the salvage therapies, such as the matching sibling donor HSCT in older patients, the unrelated or haploidentical donor HSCT in younger patients could be justified.

However, the cohort size of the current study was small. In addition, to extend follow-up, we need to expand the patient cohort in future studies. We should explore strengthening this protocol to improve the CR rate, such as the increasing dose of E-PAG to 100mg/d, or in combination with granulocyte colony-stimulating factor.

As mentioned above, our findings showed that E-PAG (75mg/d) combined with r-ATG-based IST was effective in East Asian populations. The baseline percentage of reticulocyte, ARC, RDW-CV, and ALC could be used as predictors of efficacy. It is of great significance to further expand the patient population and conduct prospective randomized controlled studies to clarify the optimal dosage, course, and response procedure of E-PAG.

## Data Availability Statement

The raw data supporting the conclusions of this article will be made available by the authors, without undue reservation.

## Ethics Statement

The studies involving human participants were reviewed and approved by The First Affiliated Hospital of Nanjing Medical University Ethic Committee. The patients/participants provided their written informed consent to participate in this study.

## Author Contributions

RL and JZ collected data, analyzed data, and wrote the paper. ZL, XC, QL, YY, SL, and JJ collected data and analyzed data. GH designed the study, performed experiments, analyzed data, and wrote the paper. JL supported experiments. All authors contributed to the article and approved the submitted version.

## Conflict of Interest

The authors declare that the research was conducted in the absence of any commercial or financial relationships that could be construed as a potential conflict of interest.

## Publisher’s Note

All claims expressed in this article are solely those of the authors and do not necessarily represent those of their affiliated organizations, or those of the publisher, the editors and the reviewers. Any product that may be evaluated in this article, or claim that may be made by its manufacturer, is not guaranteed or endorsed by the publisher.
