# Supplementary material for: Predicting Response of Severe Aplastic Anemia to Rabbit-Antithymocyte Immunoglobulin Based Immunosuppressive Therapy Combined With Eltrombopag
Source: Front Immunol. 2022 May 26;13:884312. doi: 10.3389/fimmu.2022.884312 (PMC9204341; doi:10.3389/fimmu.2022.884312)
Supplement: Supplementary file 1 [file Table_1.docx]

**Supplementary Table 1 Baseline clinical and laboratory characteristics of patients with different response^*^**

| **Characteristics** | **Responders**  **(n=44)** | **Non-responders (n=14)** | ***Value P*** |
| --- | --- | --- | --- |
| **Age, years** | **38 (18-71)** | **50.5 (24-74)** | **0.068** |
| **Male sex** | **24 (55%)** | **4 (29%)** | **0.090** |
| **Severity: SAA** | **34 (77%)** | **6 (43%)** | **0.036** |
| **Time from diagnosis to treatment, d** | **16 (1-741)** | **18 (3-1161)** | **0.84** |
| **RBC, ×10^12^/L** | **1.9 (1.2-4.1)** | **2.0 (1.0-2.5)** | **0.74** |
| **Hb, g/L** | **63 (44-128)** | **60 (37-85)** | **0.37** |
| **PLT, ×10^9^/L** | **9 (2-40)** | **6 (4-19)** | **0.26** |
| **ANC, ×10^9^/L** | **0.4 (0-4.0)** | **0.1 (0-0.7)** | **0.050** |
| **ALC, ×10^9^/L** | **1.1 (0.1-2.8)** | **0.8 (0.1-2.0)** | **0.043** |
| **ARC, ×10^9^/L** | **17.74 (0.92-97.92)** | **5.80 (0.45-30.35)** | **0.001** |
| **Reticulocyte, %** | **0.95 (0.06-5.73)** | **0.39 (0.02-1.51)** | **0.002** |
| **RDW-CV, %** | **14.8 (11.2-23.4)** | **12.6 (10.8-19.9)** | **0.134** |
| **CD3, %** | **81.0 (27.6-93.3)** | **84.2 (67.0-89.9)** | **0.57** |
| **CD4, %** | **43.0 (11.4-74.9)** | **49.5 (34.8-67.5)** | **0.24** |
| **CD8, %** | **27.3 (8.9-65.6)** | **21.7 (14.8-41.0)** | **0.39** |
| **Treg, %** | **1.9 (0-11.7)** | **1.0 (0-5.6)** | **0.67** |
| **Ferr, ng/ml** | **579.2**  **(47.0-2377.0)** | **1015.0**  **(156.8-5597.5)** | **0.070** |
| **PNH clone** | **2 (5%)** | **2 (14%)** | **0.52** |
| **Infection before IST** | **14 (32%)** | **9 (64%)** | **0.031** |

^*^Data represented the number (%) or median (range). SAA: severe aplastic anemia; RBC: red blood cell; Hb: hemoglobin; PLT: platelet; ANC: absolute neutrophil count; ALC: absolute lymphocyte count; ARC: absolute reticulocyte count; RDW-CV: red cell distribution width - coefficient of variation; Treg: regulatory T cell; Ferr: ferritin; PNH: paroxysmal nocturnal hemoglobinuria; IST: immunosuppressive therapy.

**Supplementary Table 2 Clinical results of patients with detectable somatic mutations at baseline**

| Age (years) | Gene mutations | variant allele frequency | Time to PR (months) | Time to CR (months) | Follow-up (months) | Outcome |
| --- | --- | --- | --- | --- | --- | --- |
| 29 | *DNMT3A* | 5.1% | 1 | 3 | 33 | Relapse |
| 65 | *DNMT3A* | 4.9% | 1 | - | 19 | PR |
| 28 | *DNMT3A* | 2.9% | 1 | 3 | 19 | CR |
| 39 | *MPL* | 5.9% | 1 | 23 | 29 | CR |

PR: Partial remission; CR: Complete remission.
